# Multiple LDLR class A repeats on very‐low‐density lipoprotein receptor promote the cross‐species transmission of Semliki Forest virus

**DOI:** 10.1002/mco2.351

**Published:** 2023-08-21

**Authors:** Ruolan Hu, Maochen Li, Huahao Fan

**Affiliations:** ^1^ College of Life Science and Technology Beijing University of Chemical Technology Beijing China

## Abstract

Multiple LDLR class A (LA) repeats around LA3 promote synergistic binding to Semliki Forest virus (SFV) E1‐DIII near the 2‐fold and 5‐fold symmetry axes. Meanwhile, the multiple consecutive LAs concatemer shows approximately 1000 times higher binding affinity than that of LA3s, which can help to effectively and synergistically bind with E1‐DIII of viral envelope protein.

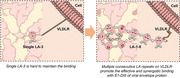

1

In a recent study published on *Cell*, Cao et al. found that multiple LDLR class A (LA) repeats around LA3 promote synergistic binding to SFV E1‐DIII near the two‐fold and five‐fold symmetry axes, explaining the specific mechanism of how the envelope protein of Semliki Forest virus (SFV) interacts with very‐low‐density lipoprotein receptor (VLDLR).^1^

SFV is an enveloped, positive‐sense, single‐stranded RNA alphavirus that normally infects neuronal cells, causes encephalitis in rodents and humans.^2^, ^3^ So far, several binding modes of alphavirus to different receptors have been reported. For example, domain 1 of LDLRAD3 can bind to VEEV by wedging the cleft created by two adjacent E2‐E1 heterodimers in a trimer spike.^4^ MXRA8 can bind to alphaviruses, such as CHIKV, ONNV, RRV, and MAYV, and has direct interactions with a surface‐exposed region across the A and B domains of CHIKV E2.^5^ The cross‐species transmission of alphavirus is usually mediated by different receptors or universal receptors on different host cells. It has been reported that the apolipoprotein receptor VLDLR is a universal receptor of several alphaviruses including SFV to infect mosquitoes, mice, horses, and humans.^6^ But the specific binding mode to the receptor and the mediated cross‐species transmission mechanism are still unclear. On April 24, 2023, Cao et al. reconstructed the binding complex of SFV to its receptor VLDLR using cryo‐electron microscopy (cryo‐EM) on *Cell*, this study will help to accelerate the development of anti‐alphavirus drugs and vaccines.^1^

Eight membrane‐distal homologous LDLR class A (LA) repeats (LA1‐8) on the extracellular structural domain of VLDLR are reported to be required for SFV's entry into the host cells.^6^ By constructing complexes of SFV virus‐like particles (VLPs) with VLDLR extracellular domain repeats, LA1‐8 was observed to bind with E1‐DIII on the SFV VLPs rather than the protruding spikes compared with other alphaviruses. The binding of LA1‐8 to SFV is flexible based on the smear density of bound LA repeats except for the region directly contacted with E1‐DIIIs. Then, individual LA repeat was expressed and their direct interactions with SFV E1‐DIII were evaluated through biolayer interferometry. LA3 was found to have the strongest binding affinity to SFV E1‐DIII, LA1, and LA2 had a modest binding affinity, LA5 showed weak binding affinity, and no binding affinity was detected among LA4, LA6, LA7, LA8 with SFV E1‐DIII (Figure 1A). Additionally, they reconstructed the symmetric viral particle model of SFV via cryo‐EM, and divided E protein on the membrane into 2f and 5f blocks (Figure 1A). Strong additional densities on 2f‐E1‐DIII but weak additional densities on 5f‐E1‐DIII were observed on the complex formed by LA3s and SFV VLPs. Almost all the residues of LA3s at the 2f blocks but only some residues of 5f were clearly visible in the density map. Severe steric clashes were found between adjacent LA3 molecules when the 2f LA3 structure was fitted into the densities at the 5f positions, indicating the occupancy of LA3 in the 5f blocks is limited. And only two separate positions could be occupied simultaneously by LA3s in the five 5f blocks.^1^

**FIGURE 1:**
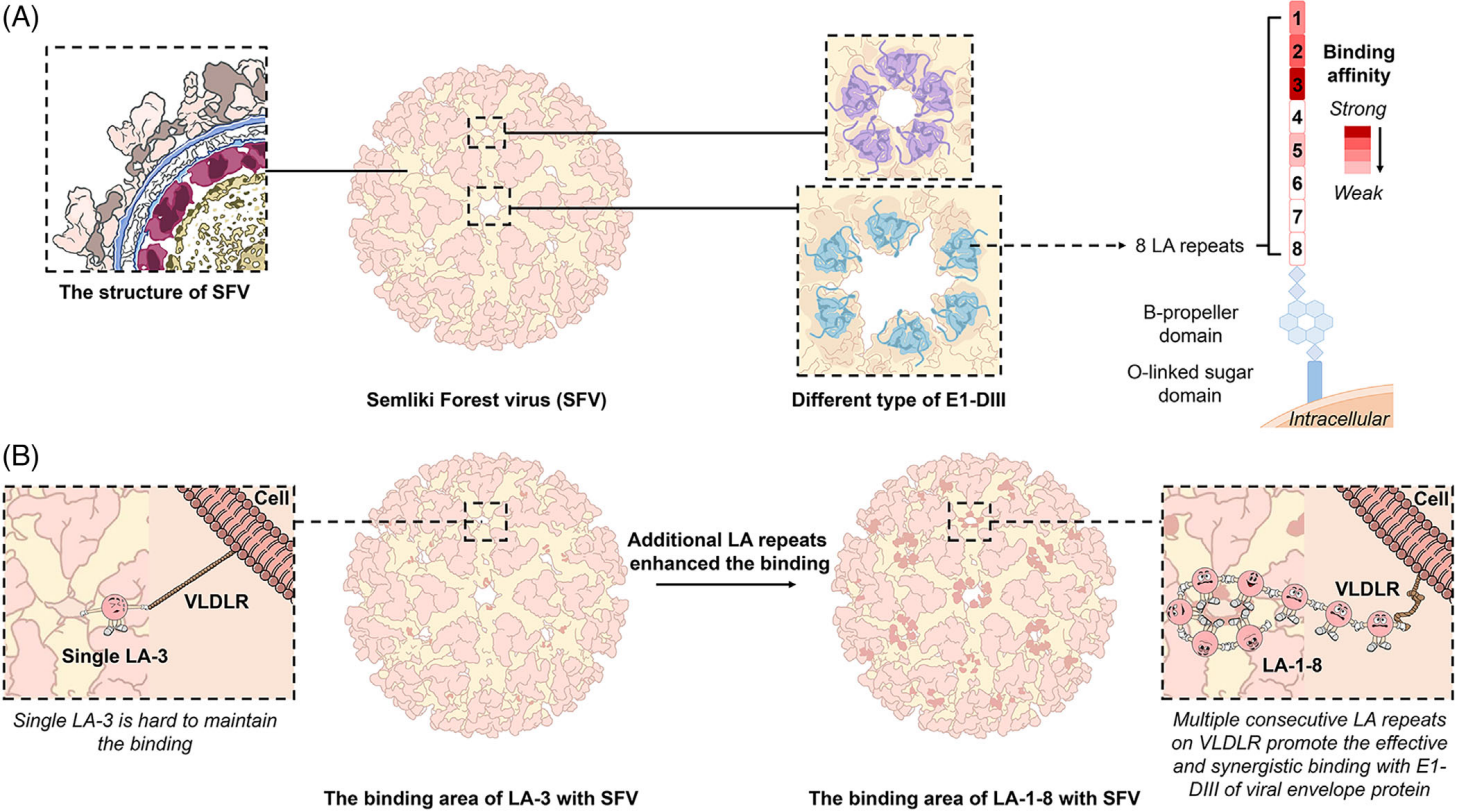
A schematic illustration of the characteristics of Semliki Forest virus (SFV) and its binding affinity to LDLR class A (LA) repeats. (A) SFV is a member of arthropod‐borne alphaviruses, and the outermost layer of the virion is a T = 4 icosahedral protein shell assembled by 240 copies of the envelope protein E1 and E2 heterodimer (shown in the left panel). The E protein on the membrane is divided into 2f and 5f blocks according to the symmetry axes, which are shown in the middle panel. The 2f‐E1‐DIII and 5f‐E1‐DIII are marked as blue and purple, respectively. The binding affinity of different LAs (LA1‐8) to E1‐DIII of SFV is shown in the right panel. (B) The binding affinity between single LA3 and E1‐DIII could not support effective viral binding to host cell as their buried surface is much smaller (only 1/3 to 1/2) than that of other alphavirus. Compared with LA3s, the multiple consecutive LAs concatemer shows approximately 1000 times higher binding affinity than that of LA3, which can help to effectively and synergistically bind with E1‐DIII of viral envelope protein.

To further investigate the interactions between LA3 and E1‐DIII, systematic mutagenesis studies were performed based on the contact residues of LA3. Mutants E117A, P129A, W132A, D135A, E137A, and D139A completely lost the ability to bind E1‐DIII, indicating that these residues have an important role in the binding between LAs and SFV VLPs. In addition, H116 and Q126 are not conserved in the other seven LAs, impairing the hydrogen bond mediated by these two residues reduced the binding affinity between LAs and SFV E1‐DIII, which implies that they may be unique residues contributing to the high binding affinity of LA3 with E1‐DIII. Moreover, the EF‐like motif of LA3 is associated with calcium‐binding, and the calcium ions could be chelated by residues W132, D135, E137, D139, D145, and E146. LA3 completely loses its binding affinity with SFV E1‐DIII after ethylene diamine tetraacetic acid (EDTA) treatment, indicating that these calcium‐binding‐related residues in LA3 are required for SFV VLPs binding. Besides, sequence comparison of LAs shows that residues W132, D135, and D139 are highly conserved in all LAs. And the binding of LAs with E1‐DIII cannot be detected when these three key residues are not coexisting on the right site of the repeats.^1^

Interestingly, the binding surface of LA3 to E1‐DIII is only 1/3 to 1/2 of counterpart in the LDLRAD3‐D1‐VEEV and MXRA8‐CHIKV complexes. As mentioned before, the binding of LA1‐8 to SFV is not fixed, suggesting there may be a combination pattern of multiple consecutive LA repeats. And the binding of LA1‐2‐Fc, LA2‐3‐Fc, LA1‐3‐Fc, LA2‐4‐Fc, LA3‐5‐Fc, LA1‐4‐Fc, LA1‐5‐Fc, LA2‐6‐Fc, and LA1‐6‐Fc to E1‐DIII was further investigated. Compared with single LAs, the simultaneous binding of multiple consecutive LAs to E1‐DIII significantly increases the binding affinity of the concatemers. Concatemers with several consecutive LA repeats, such as LA1‐6, LA1‐5, and LA1‐8, have approximately 1000 times higher binding affinity than that of LA3 (Figure 1B). Meanwhile, the binding affinity of the concatemers to E1‐DIII is not significantly affected even if the functional LA3 amino acid sites mutate, revealing a high binding affinity tolerance in the concatemers containing multiple LAs.^1^

What is more, different bound LAs at the 5f positions show similar binding patterns. Different from the single LA3 binding mode, consecutive LAs bound to E1‐DIII in the LAs concatemer all rotate about 16° around the pivot axis of the calcium‐binding site to avoid mutual spatial blockage, and thus to facilitate synergistic binding of E1‐DIII. Although most interactions between LAs concatemers and E1‐DIII are disrupted during rotation, the key sites, such as W132, D135, E137, and D139, still maintain good interactions with E1‐DIII. And the synergistic interaction among LAs allows multiple consecutive LA repeats to bind E1‐DIII simultaneously, compensating for the loss of noncritical interactions on individual LAs and greatly enhancing the viral binding to the receptor (Figure 1B).^1^

Although most amino acid sequences of VLDLR LAs in different species are not consistent, their key residues W132, E137, and D139 responsible for binding remain highly conserved.^1^ And the unique synergistic binding pattern of VLDLR's consecutive LAs makes itself highly tolerant to the sequence differences of LAs in different species during virus infection, thus enabling the cross‐species transmission of the SFV. Since the LDL family to which VLDLR belongs is the entry receptor for many important human viruses, including Rift Valley fever virus,^6^ this study may help to elucidate how these receptors specifically interact with viruses, especially the alphavirus. Besides, there are few reports on the universal receptors that mediate virus spread across distinctly different species, these unique mechanisms of VLDLR in mediating cross‐species transmission may provide important insights to people.

## AUTHOR CONTRIBUTIONS

H.F. designed the research; H.F., R.H., and M.L. read the papers and analyzed the data; R.H. and H.F. wrote and revised the manuscript. All authors have read and approved the final manuscript.

## CONFLICT OF INTEREST STATEMENT

All the authors declare no conflict of interest.

## ETHICS APPROVAL

Not applicable.

## Data Availability

Not applicable.
